# A Decolorization Technique with Spent “Greek Coffee” Grounds as Zero-Cost Adsorbents for Industrial Textile Wastewaters

**DOI:** 10.3390/ma5112069

**Published:** 2012-10-25

**Authors:** George Z. Kyzas

**Affiliations:** Department of Petroleum and Natural Gas Technology, Technological Educational Institute of Kavala, Kavala GR-65404, Greece; E-Mail: georgekyzas@gmail.com; Tel./Fax: +30-231-085-8607.

**Keywords:** industrial textile wastewaters, coffee wastes, batch studies, adsorption, dyes, decolorization

## Abstract

In this study, the decolorization of industrial textile wastewaters was studied in batch mode using spent “Greek coffee” grounds (COF) as low-cost adsorbents. In this attempt, there is a cost-saving potential given that there was no further modification of COF (just washed with distilled water to remove dirt and color, then dried in an oven). Furthermore, tests were realized both in synthetic and real textile wastewaters for comparative reasons. The optimum pH of adsorption was acidic (pH = 2) for synthetic effluents, while experiments in free pH (non-adjusted) were carried out for real effluents. Equilibrium data were fitted to the Langmuir, Freundlich and Langmuir-Freundlich (L-F) models. The calculated maximum adsorption capacities (Q_max_) for total dye (reactive) removal at 25 °C was 241 mg/g (pH = 2) and 179 mg/g (pH = 10). Thermodynamic parameters were also calculated (ΔH^0^, ΔG^0^, ΔS^0^). Kinetic data were fitted to the pseudo-first, -second and -third order model. The optimum pH for desorption was determined, in line with desorption and reuse analysis. Experiments dealing the increase of mass of adsorbent showed a strong increase in total dye removal.

## 1. Introduction

Wastewaters discharged from dye-houses are one of the biggest contributors to aquatic pollution. The most studied dye classes, in dye bearing effluent treatment, are reactive and basic [1]. However, the largest amount of the dye loss, from the dyeing process to the effluent, is estimated to be 10%–50% for reactive dyes [2]; therefore, it is necessary firstly to treat/remove these reactive dyes. Furthermore, wastewaters containing dyes are very difficult to be treated, since the dyes are recalcitrant organic molecules, resistant to aerobic digestion, and stable to light, heat and oxidizing agents [3].

The dyeing process of cotton textiles using reactive dyes involves unit operations, such as (1) desizing, (2) scouring, (3) bleaching, (4) dyeing and (5) finishing [4]. The waste streams from each individual sub-operation are collected in an “equalization tank”, where they are mixed and homogenized. In particular, the wastewater produced by the dyeing bath-reactor contains hydrolyzed reactive dyes, dyeing auxiliaries and electrolytes (60–100 g/L of NaCl and Na_2_CO_3_). The latter are responsible for the high saline content of the wastewater, which exhibits high pH values (10–11) [4]. In a typical dyeing procedure using reactive dyes, 0.6–0.8 kg NaCl, 30–60 g dyestuff, and 70–150 L water are required for the dyeing of 1 kg of cotton [5]. The large volume of colorized effluents, after the dyeing process, has to be then treated in some manner. Additionally, the research on textile effluent decolorization has often focused on reactive dyes for three main reasons [4]: (1) Reactive dyes represent an increasing market share, because they are used to dye cotton fibers, which makes up about half of the world’s fiber consumption; (2) a large fraction, typically around 30% of the applied reactive dyes, is wasted due to the dye hydrolysis in an alkaline dye bath; (3) conventional wastewater treatment plants have a low removal efficiency for reactive and other anionic soluble dyes, which leads to colored waterways [5,6].

A typical effluent treatment is broadly classified into preliminary, primary, secondary, and tertiary stages [1,6,7]. The preliminary stage includes equalization and neutralization [7]. The primary stage involves screening, sedimentation, flotation, and flocculation. The secondary stage reduces the organic load and facilitates the physical/chemical separation (biological oxidation), while the tertiary stage is focused on decolorization [7]. In the latter stage, the adsorption onto activated carbon is broadly used to limit the concentration of color in effluents [8]. Adsorption has been applied either in a single mode, mainly for dyes removal from synthetic wastewaters, or in a combinational mode for total cleaning of real dyeing wastewaters.

Recently, many works have been studied for the development of effective and low-cost adsorbents. A published study of Crini [9] reported in details the main low-cost and non-conventional adsorbents for dyes removal (natural materials, biosorbents, waste materials from industry and agriculture). Some of the reported adsorbents include clay materials (bentonite, kaolinite), zeolites, siliceous material (silica beads, alunite, perlite), agricultural wastes (bagasse pith, maize cob, rice husk and coconut shells) [9,10,11], industrial waste products (waste carbon slurries and metal hydroxide sludge) [9,12], biosorbents (chitosan, peat and biomass) and others (starch, cyclodextrin and cotton) [4,9]. However, there is a lack of literature dealing with the possible application of coffee residues as adsorbents (*i.e.*, for metals) [13,14], and in particular as dye adsorbents [15,16,17,18].

In general, “coffee residues” are generally called the solid wastes discarded from the extraction process of instant coffee manufacturing, and the final residues originated from cafeterias. In recent years, the instant coffee industry has experienced a constant growth as instant coffee has become one of the most popular kinds of coffee, drunk by millions of people around the world. As a consequence, large amounts of coffee grounds, which are the solid residues obtained during the processing of coffee powder with hot water or steam to prepare instant coffee, have been generated worldwide (6,000,000 t/yr) [19].

However, the screening of literature dealing with the “textile wastewaters” leads to many works on dye removal by adsorption from “synthetic effluents/mixtures” [9], while very few on “real (industrial) dyeing effluents/mixtures” [4,11,12].

The novelty of this study is the processing (decolorization) of real effluent originated directly from the dyeing bath, just before entering the equalization tank (so, only a small fraction (~10%) of the entire discharge volume will be treated, which means saving money and energy) [5] using industrial wastes (coffee residues) as adsorbents; the coffee wastes used were non-chemically modified. There is a high potential in this attempt given that despite the possible limited adsorption capacity of these materials, their cost is near to zero (the coffee wastes were only dried at ambient temperature and used for the adsorption experiments.

In the current work, the decolorization of real textile wastewaters and synthetic dye mixtures was performed in batch mode, studying the effect of: (1) pH on adsorption; (2) agitation rate; (3) initial total dye concentration; (4) contact time; (5) temperature in equilibrium (isotherms); (6) pH on desorption, and (7) reuse in sequential cycles of adsorption-desorption.

## 2. Materials and Methods

### 2.1. Adsorbents—Spent “Greek coffee” Grounds (COF)

The low-cost adsorbents used were non-modified industrial wastes of a special variety of coffee namely “Greek coffee” (abbreviated hereafter as COF) from cafeterias. The coffee residues were not chemically treated (without any modification to further improve their adsorptive ability), but just only washed with distilled water to remove dirt and color, and dried at 105 °C for 5 h in a convection oven. The residues used were in powder form (475–525 μm) after sieving. All experiments were repeated four times and the experimental points (in adsorption figures) display the average of the data found. This was realized in order to diminish the experimental faults.

### 2.2. Adsorbates—Dyeing Mixtures

**Figure 1:**
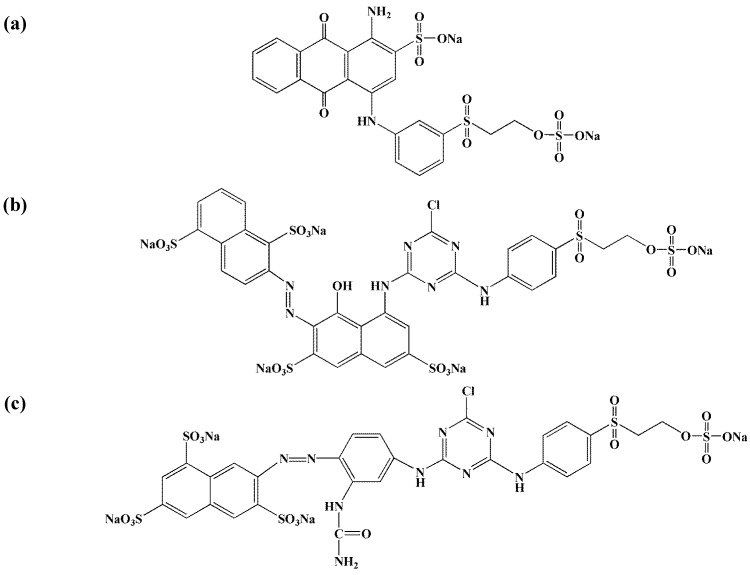
Chemical structures of reactive dyes used: (**a**) Remazol Brillant Blue RN (RB); (**b**) Remazol Red 3BS (RR); (**c**) Remazol Yellow Gelb 3RS (RY).

The real textile samples of wastewater from the dyeing reactor were kindly supplied by “Dyeing-Finishing mills of Thessaloniki, Greece” and composed of three reactive dyes: Remazol Red 3BS (abbreviated hereafter as RR, C_31_H_19_ClN_7_Na_5_O_19_S_6_, MW = 1136.31 g/mol, λ_max_ = 588 nm, 58% w/w), Remazol Yellow gelb 3RS 133% (abbreviated hereafter as RY, C_28_H_20_ClN_9_Na_4_O_16_S_5_, MW = 1026.25 g/mol, λ_max_ = 419 nm, 60% w/w), and Remazol Blue RN (abbreviated hereafter as RB, C_22_H_16_N_2_Na_2_O_11_S_3_, MW = 626.54 g/mol, λ_max_ = 541 nm, 56% w/w). The chemical structures of all dyes used are given in Figure 1.

Additionally, the same dyes in powder-form were purchased by DyStar and used as reagents for the preparation of synthetic dyeing samples. It should be stressed that the commercial dyes were supplied as a mixture of the active material and an inert product. This was taken into account and the concentrations in liquid and solid phase, which are presented in experimental results (Section 3), are actual (true) concentrations.

### 2.3. Characterization

Characterization was realized to evaluate the surface chemistry and morphology of the low-cost materials used. The point of zero charge (PZC) was evaluated according to titration procedures described in literature [17,18,20]. Three aqueous solutions of different pH values (3, 6 and 11) were prepared. Several amounts of coffee wastes were added to 20 mL of each solution to prepare 0.05%, 0.1%, 0.5%, 1.0%, 3.0%, 7.0% and 10.0% w/w coffee/solutions. The aqueous suspensions containing different amounts of the adsorbent were let to equilibrate for 24 h under agitation (140 rpm) at 25 °C. The pH of each solution was then measured. The PZC was determined as the converging pH value from the pH *versus* adsorbent mass curve.

The determination of surface functional groups was based on the Boehm titration method [21]. Aqueous solutions of NaHCO_3_ (0.10 mol/L), Na_2_CO_3_ (0.05 mol/L), NaOH (0.10 mol/L), and HCl (0.10 mol/L) were prepared. 50 mL of these solutions were added to vials containing 1 g of dry coffee samples, let to be shaken (140 rpm) until equilibrium (24 h), and then filtered. Five blank solutions (without the adsorbent) were also prepared. In this way, the number of the basic sites was calculated from the amount of HCl that reacted with the coffee adsorbents. The various free acidic groups were derived using the assumption that NaOH neutralizes carboxyl, lactone and phenolic groups, Na_2_CO_3_ neutralizes carboxyl and lactone and NaHCO_3_ neutralizes only carboxyl groups. The excess of base or acid was then determined by back titration using NaOH (0.10 mol/L) and HCl (0.10 mol/L) solutions [20].

Scanning electron microscopy (SEM) observations of the prepared particles were carried out using a JEOL JMS-840A scanning microscope equipped with an energy-dispersive X-ray (EDX) Oxford ISIS 300 micro-analytical system. All of these surfaces were coated with a thin layer of carbon black to avoid charging under the electron beam.

The surface area was also determined with a Micromeritics BET (Brunauer, Emmett and Teller) surface area analyzer, model TriStar 3000, by means of adsorption of ultra pure nitrogen.

### 2.4. Adsorption-Desorption Experiments

Before the adsorption experiments, the real dyeing effluent supplied was analyzed (based on methods described in Section 2.5) and the concentrations of each dye were found to be 197 mg/L RR, 223 mg/L RY, and 280 mg/L RB (RR, 28%; RY, 32%; RB, 40%). Based on this, the synthetic effluent (mixture) was composed of the same dye concentrations along with 1 mol/L NaCl ionic strength.

The effect of pH was conducted by mixing 1 g/L of adsorbent with 50 mL of both synthetic and real sample (total dye concentration 700 mg/L: 197 mg/L RR, 223 mg/L RY, and 280 mg/L RB). The pH value, ranging between 2 and 12, was kept constant throughout the adsorption process by micro-additions of HNO_3_ (0.01 mol/L) or NaOH (0.01 mol/L). The suspension was shaken for 24 h (agitation rate = 140 rpm) into a water bath to control the temperature at 25 °C (Julabo SW-21C). The optimum pH found for synthetic dyeing mixture was pH = 2 (see Section 3.2).

Kinetic experiments were performed by mixing 1 g/L of COF with 50 mL of real and synthetic dyeing mixtures (total dye concentration 700 mg/L). The suspensions were shaken for 24 h at non-adjusted (natural) pH (~10) in water bath at 25 °C (agitation rate = 140 rpm). Samples were collected at fixed intervals (5–30 min, 1–24 h). The following three equations (pseudo-first order, Equation (1); pseudo-second order, Equation (2); pseudo-third order, Equation (3)) were selected to fit the experimental kinetic data [22]:

(1) $${\text{C}}_{t}={\text{C}}_{0}-({\text{C}}_{0}-{\text{C}}_{e})(1-{\text{e}}^{-{\text{k}}_{1}\text{t}})$$

(2) $${\text{C}}_{t}={\text{C}}_{0}-({\text{C}}_{0}-{\text{C}}_{e})(1-\frac{1}{1+{\text{k}}_{2}\text{t}})$$

(3) $${\text{C}}_{t}={\text{C}}_{0}-({\text{C}}_{0}-{\text{C}}_{e})(1-\frac{1}{{\left(1+2{\text{k}}_{3}\text{t}\right)}^{1/2}})$$

Where k_1_, k_2_, k_3_ (min^−1^) are the rate constants for the pseudo-first, -second and -third order adsorption kinetic model, respectively, and C_0_, C_t_, C_e_ (mg/L) are the initial, transient and equilibrium total concentrations of dye in the aqueous solution, respectively.

The effect of total initial dye concentration (synthetic samples) on equilibrium was observed by mixing 1 g/L of COF with 50 mL of dye solutions of different initial dye concentrations (0–1000 mg/L). Given that the concentrations of each dye in the real dyeing sample were analyzed and found to be 197 mg/L RR, 223 mg/L RY, and 280 mg/L RB, the same proportion was adopted to the preparation of synthetic effluent for the experiments of the effect of total initial dye concentration. The suspensions were shaken for 24 h at pH = 2 (optimum pH found) and pH = 10 (natural/free pH value of real textile effluent) in water bath at 25 °C (agitation rate = 140 rpm). The equilibrium data resulted were fitted to the Langmuir (Equation (4)) [23], Freundlich (Equation (5)) [24], and Langmuir-Freundlich (L-F) (Equation (6)) isotherm model [25], expressed by the following equations:

(4) $${\text{Q}}_{e}=\frac{{\text{Q}}_{max}{\text{K}}_{L}{\text{C}}_{e}}{1+{\text{K}}_{L}{\text{C}}_{e}}$$

(5) $${\text{Q}}_{e}={\text{K}}_{F}{\text{C}}_{e}^{1/\text{n}}$$

(6) $${\text{Q}}_{e}=\frac{{\text{Q}}_{max}{\left({\text{K}}_{LF}{\text{C}}_{e}\right)}^{\text{b}}}{1+{\left({\text{K}}_{LF}{\text{C}}_{e}\right)}^{\text{b}}}$$

Where Q_e_ (mg/g) is the equilibrium dye concentration in the solid phase; Q_max_ (mg/g) is the maximum amount of adsorption; K_L_ (L/mg) is the Langmuir adsorption equilibrium constant; K_F_ (mg^1−1/n^ L^1/n^g^−1^) is the Freundlich constant representing the adsorption capacity, n is the constant depicting the adsorption intensity, K_LF_ (L/mg)^1/b^ is the L-F constant, and b is the L-F heterogeneity constant. The amount of total dye uptake at equilibrium Q_e_ (mg/g) was calculated using the mass balance equation (Equation (7)):

(7) $${\text{Q}}_{e}=\frac{({\text{C}}_{0}-{\text{C}}_{e})\text{V}}{\text{m}}$$

where m (g) is the mass of adsorbent; V (L) the volume of adsorbate; C_0_ and C_e_ (mg/L) are the initial and equilibrium dye concentrations in the liquid phase, respectively.

Based on the equilibrium data of isotherms, thermodynamic parameters were calculated. The Gibbs free energy change, ΔG^0^ (kJ/mol), of the adsorption process is related to the equilibrium constant (K_c_) by the Van’t Hoff equation (Equation (8)) [26]:

(8) $$\text{∆}{\text{G}}^{0}=-\text{R T ln}({\text{K}}_{c})$$

where R (=8.314 J/mol·K) is the universal gas constant.

The constant K_c_ can be calculated from Equation (9):

(9) $${\text{K}}_{c}=\frac{{\text{C}}_{s}}{{\text{C}}_{e}}$$

where C_s_ (mg/L) is the amount adsorbed on solid at equilibrium.

It is also related to the change in entropy (ΔS^0^, kJ/mol·K) and the heat of adsorption (ΔH^0^, kJ/mol) at a constant temperature T (K), as follows (Equation (10)):

(10) $$\text{∆}{\text{G}}^{0}=\text{∆}{\text{H}}^{0}-\text{T∆}{\text{S}}^{0}$$

From Equations (8)–(10):

(11) $$\text{ln}({\text{K}}_{c})=\frac{1}{\text{T}}(\frac{-\text{∆}{\text{H}}^{0}}{\text{R}})+\frac{1}{\text{R}}\text{∆}{\text{S}}^{0}$$

The values of ΔH^0^ and ΔS^0^ were calculated from the slop and intercept of the plot between ln(Kc) *versus* (1/T).

In the case of real samples, where the initial dye concentration cannot be varied, the key-factor is the determination of the mass of adsorbent (which is able to decolorize sufficiently the dyeing solutions). To determine the effect of the mass of adsorbent, experiments were carried out varying the dosage (1–10 g/L) and keeping constant all the other parameters: pH (free) = 10; total dye concentration 700 mg/L (as calculated from dye analysis), 25 °C; 140 rpm; 24 h.

After adsorption experiments (where COF adsorbents were exposed to 700 mg/L of real textile wastewaters; 25 °C; pH~10; 24 h; 140 rpm), the samples were collected and filtered, using fixed pore-sized membranes. A small fraction of the dye (1%–2%) and the adsorbent (1%) were retained on the filter membrane; these small variations due to filtration were neglected. To determine the optimum desorption pH value of the dye-loaded adsorbents, experiments were realized by mixing the collected (after adsorption) amount of dye-loaded adsorbents (0.05 g) with aqueous solutions of 50 mL (same volume as in the adsorption step) over a pH range between 2 and 12, at 25 °C for 24 h (agitation rate = 140 rpm). To determine the reusability of the COF adsorbents, 10 sequential adsorption-desorption cycles were repeated, using the same adsorbents and following the experimental procedures described above in the optimum conditions found.

### 2.5. Analysis

The dye content of samples was measured spectrophotometrically (UV-Vis, model U-2000, Hitachi). The effect of pH and aquatic environment on both the calibration curves and λ_max_ (maximum wavelength of each dye) were studied, but no significant deviation was observed. For synthetic dyeing mixtures, the dye content (expressed in units of concentration, mg/L) was calculated as described below:

The absorbance of the mixture was measured in the three wavelengths of maximum absorbencies of dyes (λ_max,RR_, λ_max,RB_, λ_max,RY_). The molar absorptivity/extinction coefficients E (L·mol^−1^·cm^−1^) for the three dyes was calculated from the Lambert-Beer law (A=E·Z·C), where Z (cm) is the path length of the cell. The resulting 3 × 3 equation system (expressed by Equation (12–14)) gives the residual concentration of each reactive dye [27]:

(12) $${\text{A}}_{\lambda 1}={\text{E}}_{\lambda 1,\text{RY}}\cdot \text{Z}\cdot {\text{C}}_{RY}+{\text{E}}_{\lambda 1,\text{RB}}\cdot \text{Z}\cdot {\text{C}}_{RB}+{\text{E}}_{\lambda 1,\text{RR}}\cdot \text{Z}\cdot {\text{C}}_{RR}$$

(13) $${\text{A}}_{\lambda 2}={\text{E}}_{\lambda 2,\text{RY}}\cdot \text{Z}\cdot {\text{C}}_{RY}+{\text{E}}_{\lambda 2,\text{RB}}\cdot \text{Z}\cdot {\text{C}}_{RB}+{\text{E}}_{\lambda 2,\text{RR}}\cdot \text{Z}\cdot {\text{C}}_{RR}$$

(14) $${\text{A}}_{\lambda 3}={\text{E}}_{\lambda 3,\text{RY}}\cdot \text{Z}\cdot {\text{C}}_{RY}+{\text{E}}_{\lambda 3,\text{RB}}\cdot \text{Z}\cdot {\text{C}}_{RB}+{\text{E}}_{\lambda 3,\text{RR}}\cdot \text{Z}\cdot {\text{C}}_{RR}$$

Where A_λ1_, A_λ2_, and A_λ3_ are the absorbencies of the mixture at λ_max_ of RY dye (λ_1_ = 588 nm), RR (λ_2_ = 541 nm), and RB (λ_3_ = 419nm), respectively. Ε_λ1,RY_, Ε_λ1,RB_ , Ε_λ1,RR_ are the absorbance coefficients of pure dyes RY, RB, RR at the λ_max_ of RY dye. Ε_λ2,RY_, Ε_λ2,RB_, Ε_λ2,RR_ are the absorbance coefficients of pure RY, RB, RR at the λ_max_ of RR dye. Ε_λ3,RY_, Ε_λ3,RB_ , Ε_λ3,RR_ are the absorbance coefficients of pure RY, RB, RR at the λ_max_ of RB dye.

To obtain a direct application to the legislation limits, apart from concentration units (mg/L), the residual total dye concentration was expressed in ADMI units. Briefly, this method involves measuring the absorbance of samples [7,28], after filtration at sets of 30 wavelengths (usually 400–700 nm) depending on the accuracy required to generate the CIE (Commission International de l’Eclairage) Tristimulus Values, X, Y and Z. Tristimulus values for samples are X_s_, Y_s_, and Z_s_; for standards are X_r_, Y_r_, and Z_r_; and for distilled water are X_c_, Y_c_, Z_c_. These are converted by the use of published tables to values called Munsell values. Munsell values for samples are V_xs_, V_ys_, V_zs_; for standards V_xr_, V_yr_,V_zr_ ; and for distilled water V_xc_, V_yc_,V_zc_. From the Munsell values the Adams-Nickerson Color Difference (DE) is calculated from the Equation (15):

(15) $$\text{DE}={\left[{\left(0.23\text{∆}({\text{V}}_{y})\right)}^{2}+{\left(\text{∆}({\text{V}}_{x}-{\text{V}}_{y})\right)}^{2}+{\left(0.4\text{∆}({\text{V}}_{y}-{\text{V}}_{z})\right)}^{2}\right]}^{1/2}$$

where:

(16) $$\text{∆}({\text{V}}_{y})={\text{V}}_{ys}-{\text{V}}_{yc}$$

(17) $$\text{∆}({\text{V}}_{x}-{\text{V}}_{y})=({\text{V}}_{xs}-{\text{V}}_{ys})-({\text{V}}_{xs}-{\text{V}}_{yc})$$

(18) $$\text{∆}({\text{V}}_{y}-{\text{V}}_{z})=({\text{V}}_{ys}-{\text{V}}_{zs})-({\text{V}}_{yc}-{\text{V}}_{zc})$$

The DE values of a series of APHA platinum-cobalt standards is plotted against the corresponding ADMI values to give a calibration plot and the DE value of samples read against this plot to obtain the ADMI value of the sample.

## 3. Results and Discussion

### 3.1. Characterization

The composition of COF was determined [29] as 44% water, 12% protein, 14% lipids, 25% carbohydrates and 5% ash. According to Boehm method [21], the functional groups at the surface of COF were carboxylic 0.97 mmol/g_COF_ and basic 0.93 mmol/g_COF_, followed by phenolic 0.14 mmol/g_COF_ and 0.11 lactonic mmol/g_COF_.

**Figure 2:**
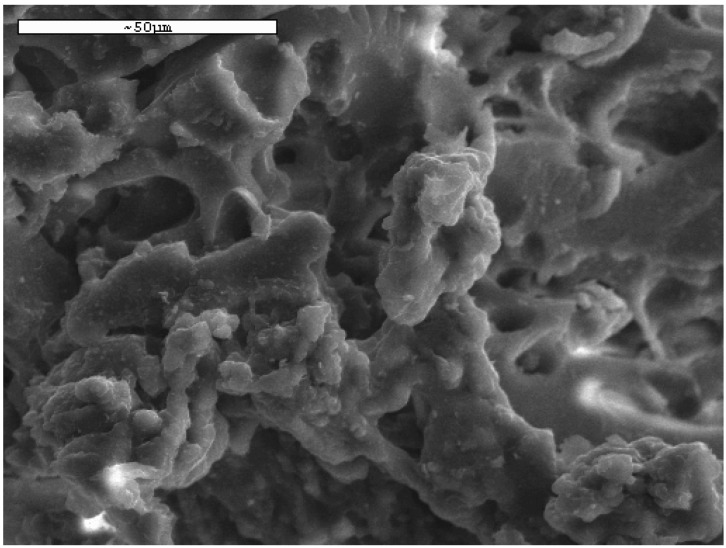
Scanning Electron Micrograph of the “Greek coffee” grounds (COF).

Figure 2 presents the morphology of COF according to SEM micrographs taken. It is obvious that its surface was not smooth, but full of cavities. These cavities can be characterized as channels onto the surface of COF instead of pores [30], given the small surface area calculated from BET analysis (~2.3 m^2^/g).

The energy dispersive X-ray microanalysis (SEM/EDX) of the coffee wastes indicates mainly the presence of oxygen (64.11%) and carbon (30.59%). Also a variety of other elements (5.30%) was also determined mainly as admixtures (K, Na, Mg, P, S).

**Figure 3:**
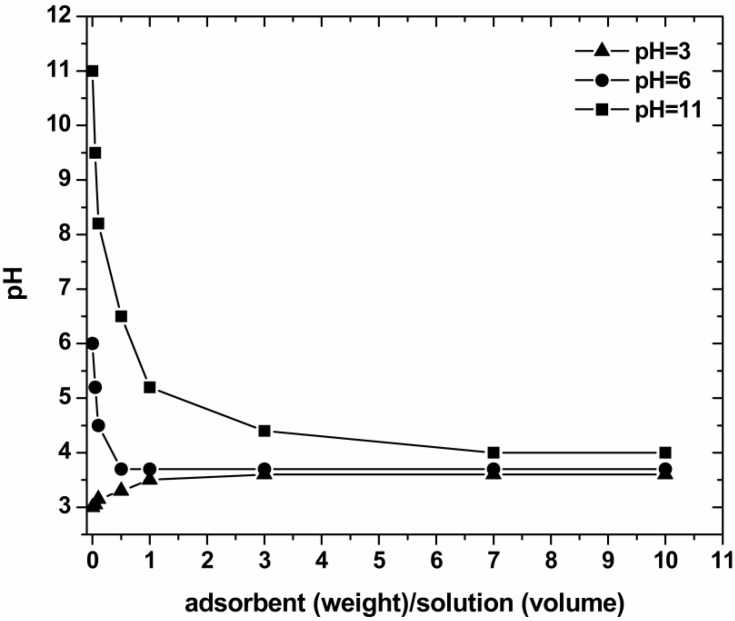
Determination of the point of zero charge (PZC) of the COF.

The experimental titration curves for PZC determination are presented in Figure 3. These results indicated the PZC values in the range of 3.3–3.5. For pH values above of 3.5, a predominant negatively charged surface of COF thusoccurred. At lower pH values, the surface charge may get mainly positively charged. The aforementioned consideration was proved and extensively explained in line with the adsorption mechanism of dyes onto coffee surface in previously published works [17,18,30,31].

### 3.2. Effect of pH

**Figure 4:**
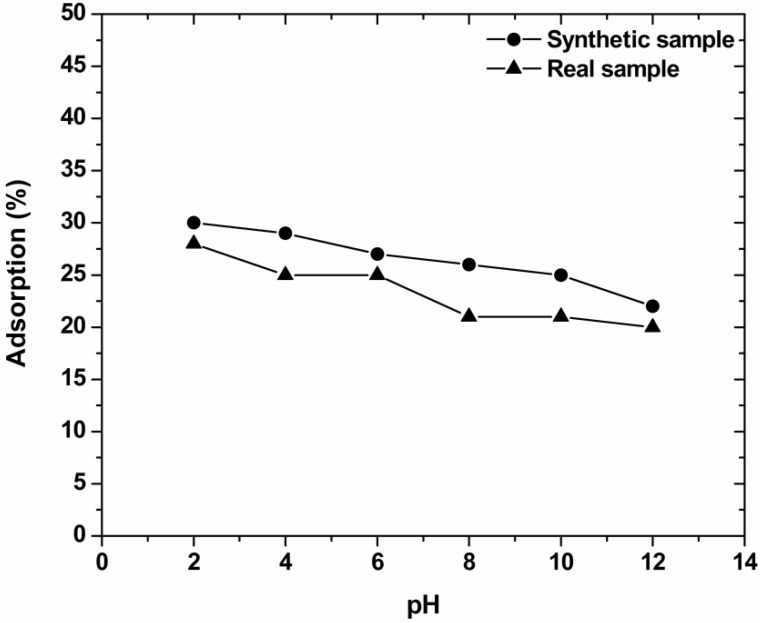
Effect of pH on decolorization of real and synthetic dyeing solutions with COF (pH = 2–12; 1 g/L adsorbent; 700 mg/L total dye concentration; T = 25 °C; 140 rpm; 24 h contact).

One of the most important factors, affecting the capacity of an adsorbent in wastewater treatment, is pH. The effect of pH on decolorization from COF is shown in Figure 4. Firstly, in order to examine whether the structure of each dye used is stable (the same before and after adsorption), a simple experiment was performed. It was achieved using a comparison of the UV-Vis spectrum before the beginning of adsorption (without adsorbent, just only aqueous solution at the particular alkaline value pH = 12), and the respective spectrum taken from the dye solution after the end of process (again in the same pH). No significant change was observed, suggesting that the dyes structure seemed to be unchanged. In any case, the existence of auxiliaries in real samples makes more difficult the stability of dye molecules.

From a first point of view, all curves presented the same trend both for real and synthetic dyeing wastewaters. The maximum dye removal percentage was observed for adjusted pH = 2 (synthetic, 30%; real, 28%). It then becomes obvious that the percentages of total dye removal (quantitatively) were lower in the case of real effluents (see Figure 4), due to the presence of dyeing auxiliaries [5]. However, the change in percentages between real and synthetic effluents was only 2%. In general, the higher dye uptakes were showed in acidic pH-region, due to the acidic nature of reactive dyes (this type of dyes contains sulfonate groups, as seen in Figure 1) acidic pH values [32], the surface of COF can be easily charged positively due to the excess of H^+^ groups in solution. Therefore, the positively charged sites of COF can interact with the negative sulfonate groups of reactive dyes, forming a strong bond between adsorbent and dye. The most promising finding was that the dye removal percentages of COF was changed only 5% (from 28% to 23%) at the nature pH value of the real textile effluent (~10). The dye removal percentage at even pH = 10 could be attributed to a combination of decreased electrostatic and other interactions, as van der Waals forces, hydrogen bonding and pi-pi interactions [4,30,33]. The pH of the solution in real sample was allowed to be unadjusted (free). In these strong alkaline conditions no significant pH change was observed during adsorption.

In general, the adsorption mechanism of dye onto the solid adsorbent involves different steps, as: (1) The transport of the dye molecule from the bulk solution to the adsorbent surface; (2) adsorption on the particle surface; and (3) transport within the adsorbent particle. However, the COF used as adsorbents presented complexity of their specific characteristics (such as the presence of complexing chemical groups, *etc.*). The interaction occurred and the possible adsorption mechanism is complicated because they implicate the presence of different interactions. In addition, a wide range of chemical structures (in the current study, three different dye molecules were used), pH, and salt concentrations often add to the complication. Some of the reported interactions include: (1) ion-exchange; (2) complexation; (3) coordination/chelation; (4) electrostatic interactions; (5) acid-base interactions; (6) hydrogen bonding; (7) hydrophobic interactions; (8) physical adsorption; (9) precipitation [1,2,6,9]. In the case of reactive dye removal, where the adsorption is strongly pH-dependent, the dominated interactions are influenced by the pH value of solution. Strong electrostatic forces are dominated in acidic pHs and even hydrophobic dye-dye or dye-adsorbent interactions in the whole pH range. The aforementioned mechanism is already explained in previous work [30,34].

Further adsorption experiments (kinetics, agitation rate, adsorbent’s dosage) were then performed at the natural pH value (pH ~ 10) for real samples of wastewaters, because: (1) pH = 10 is the original alkalinity of textile effluents; (2) additional cost will be avoided due to the non pH-regulation; (3) dye removal at this pH still presents only 5% decrease with respect to pH = 2 for COF (see Figure 4).

### 3.3. Kinetics

**Figure 5:**
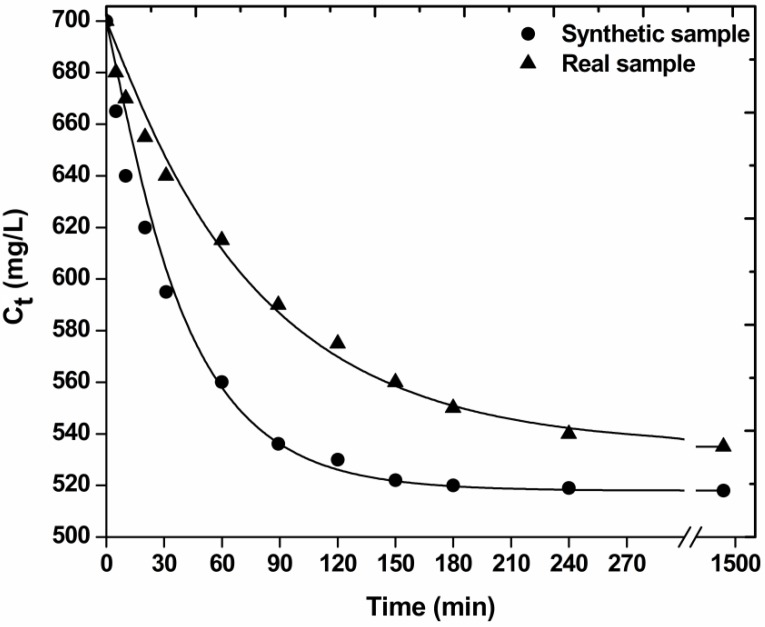
Effect of contact time on decolorization of real and synthetic dyeing solutions with COF (pH = free, 700 mg/L total dye concentration; 1 g/L adsorbent; T = 25 °C; 140 rpm; 5 min–24 h contact).

Figure 5 illustrates the effect of contact time on decolorization (dye adsorption) with the low-cost materials used. The plots (synthetic and real sample) could be divided in three zones: (1) 0–30 min, which indicated the instantaneous adsorption of dyes, suggesting rapid external diffusion and surface adsorption; (2) 30–180 min, showed a gradual equilibrium; and (3) 3–24 h, indicated the plateau of the equilibrium state [33]. It can be seen that the adsorption was rapid at the initial stage of the contact, but it gradually slowed down until the equilibrium. The fast adsorption at the initial stage can be attributed to the fact that a large number of surface sites are available for adsorption. After a lapse of time, the remaining surface sites are difficult to be occupied because of the repulsion between the solute molecules of the solid and bulk phases make it took long time to reach equilibrium [15].

Table 1 presents the kinetic parameters resulted from the fitting of the pseudo-first, -second and -third order equation to the experimental kinetic data. Based on the correlation coefficients (R^2^) exported, the best fitting (for real dyeing sample) was observed for the pseudo-first order equation (R^2^ = 0.994), while the pseudo-second (R^2^ = 0.967), and pseudo-third (R^2^ = 0.889) order equations presented enough lower coefficients. The kinetic rate was established with the parameter “k” calculated (k_1_ = 0.013 min^−1^). Comparing with the respective kinetic parameter for synthetic sample, it is clear that its decolorization “runs” faster (k_1_ = 0.021 min^−1^), which can be easily attributed to the co-existence of auxiliaries in real sample. The excess of impurities and dye auxiliaries prevents the rapid removal of dye molecules and their binding onto COF surface.

**Table 1:** Kinetic constants for the decolorization of real and synthetic effluent with COF at 25 °C.

Sample	Pseudo-first order		Pseudo-second order		Pseudo-third order	
	k_1_	R^2^	k_2_	R^2^	k_3_	R^2^
	min^−1^		min^−1^		min^−1^	
Real	0.013	0.994	0.026	0.967	0.051	0.889
Synthetic	0.021	0.994	0.031	0.966	0.058	0.877

### 3.4. Effect of Initial Dye Concentration—Isotherms (Synthetic Samples)

**Figure 6:**
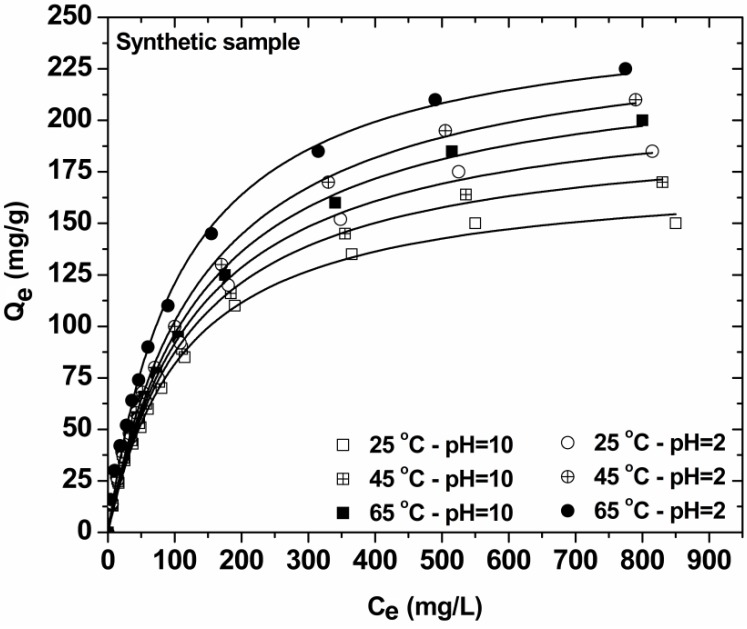
Isotherms of decolorization of synthetic dyeing solutions with COF (pH = 2, 10; 0–1000 mg/L total dye concentration; 1 g/L adsorbent; T = 25, 45, 65 °C; 140 rpm; 24 h contact).

Figure 6 presents the isotherms resulted from the decolorization of synthetic effluent at two pH values (in order to compare the Q_max_ of low-cost COF at natural and optimum pH value). Table 2 reports the maximum adsorption capacities (Q_max_) and the other isothermal parameters resulted from the fitting. The correlation coefficients (R^2^) confirmed that both L-F and Langmuir models fit the experimental data with high correlation (L-F: R^2^ ~ 0.999; Langmuir: R^2^ ~ 0.997). The Q_max_ for total dye removal at 25 °C was 241 mg/g (pH = 2) and 179 mg/g (pH = 10). The reduction of capacity was only 60 mg/g. A review paper of Sharma et al [35] presents various natural or agricultural wastes (activated carbon from agricultural sources, rice husk, fly ash, sugarcane dust, cotton waste, sludge ash, plant leaf powder, plant fibers, wood shaving, tea waste, oil palm wood, sawdust, *etc.*) which have been used as dye adsorbents. However, their adsorption capacity is ranged (2–600 mg/g). A direct comparison cannot be achieved given the different experimental conditions for each study. Also, the current study describes the application of coffee wastes (COF) to real dyeing samples and not only synthetic ones. The experimental data resulting are thus non-comparative.

**Table 2:** Equilibrium parameters for the adsorption of dyes from synthetic effluent onto COF at optimum pH = 2 and natural (non-adjusted) pH ~ 10 at 25, 45, and 65 °C.

Synthetic sample		Langmuir equation			Freundlich equation			Langmuir-Freundlich (L-F) equation			
	T	Q_m_	K_L_	R^2^	K_F_	n	R^2^	Q_m_	K_LF_	b	R^2^
	°C	mg/g	L/mg		mg^1−1/n^L^1/n^g^−1^			mg/g	(L/mg)^1/b^		
pH = 10	25	175	0.0088	0.997	11.83	2.53	0.957	179	0.0082	0.957	0.997
	45	197	0.0080	0.997	11.59	2.41	0.969	212	0.0066	0.898	0.999
	65	232	0.0071	0.997	11.44	2.27	0.980	269	0.0047	0.828	0.999
pH = 2	25	214	0.0075	0.997	11.47	2.34	0.976	241	0.0054	0.850	0.999
	45	245	0.0072	0.997	12.08	2.27	0.978	278	0.0051	0.848	0.999
	65	254	0.0091	0.997	15.85	2.43	0.975	287	0.0064	0.835	0.999

In general, the equilibrium dye uptake was affected by the initial dye concentration using constant dosage of COF (1 g/L). At low initial concentrations, the adsorption of dyes seemed to be very intense and reached equilibrium rapidly. This phenomenon indicated the possibility of the formation of monolayer coverage of dye molecules at the outer interface of COF. Furthermore, for low total dye concentrations (0–50 mg/L) the ratio of initial number of dye molecules to the available adsorption sites is low and subsequently the fractional adsorption becomes independent on initial concentration [36,37]. According to BET classification [38], I-type isotherms represents unimolecular adsorption and applies to non-porous, microporous and adsorbents with small pore sizes (not significantly better than the molecular diameter of the adsorbate). The shapes of curves (Figure 6) thus indicated that the isotherms for the adsorbent-dye systems studied were I-Type and characterized by a high degree of adsorption at low concentrations. At higher concentrations, the available adsorption sites were lower and subsequently the adsorption depended on the initial concentration of dye. As a matter of fact, the diffusion of exchanging molecules within COF particles may govern the adsorption rate at higher initial concentrations.

The effect of temperature on equilibrium is also presented through isotherms curves (Figure 6).The adsorption behavior is similar for both pH-conditions of synthetic effluents: Increasing the temperature of process from 25 to 65 °C, an increase of the adsorption capacity (dye uptake) is observed. Similar results were already published [4].

### 3.5. Thermodynamics

The parameters of ΔH^0^ and ΔS^0^ were calculated from the slop and intercept of the plot between ln(K_c_) *versus* (1/T) (R^2^ > 0.990, data not shown). These parameters, at selected total dye concentrations and all temperatures, are given in Table 3 for synthetic effluents (pH = 2 and pH = 10). The positive values of ΔH^0^ suggest the endothermic nature of the process. The negative values of ΔG^0^ (initial total dye concentrations <500 mg/L) suggest that the process is spontaneous with high preference for dye molecules. In the case of high initial total dye concentrations (C_0_ > 500 mg/L), enthalpy is positive presenting non-spontaneous behavior. Since the adsorption is endothermic, the amount adsorbed at equilibrium is increased with increasing temperature. The positive values of ΔS^0^ show the increased randomness at the solid/liquid interface. During adsorption, the coordinated water molecules (which are displaced by the dye molecules) gain more translational entropy than is lost by the dye molecules, resulting in increased randomness in the dye-adsorbent interaction [39,40]. It is well known that ionic dyes trend to aggregate in dilute solutions, leading to dimmer formation [4,41]. It is supposed that dimmer formation in solution is mainly due to hydrophobic interactions or permanent and transition dipole moments [4,41]. Although dyes are very individualistic in structure, certain broad rules are well established regarding their dimerization. The probability increases with an increase of dye concentration or ionic strength; it will decrease with temperature rising or organic solvents adding [4,41]. In the current study, the use of three reactive dyes, as well as the existence in real sample numerous of even organic auxiliaries, prevents from an obvious conclusion in this topic.

**Table 3:** Thermodynamic parameters for the decolorization of synthetic effluent with COF.

Synthetic sample	C_0_	T	Q_e_	K_c_	ΔG^0^	ΔH^0^	ΔS^0^
	mg/L	K	mg/g		kJ/mol	kJ/mol	kJ/mol K
pH = 2	20	298	13.72	2.17	−1.92	+5.79	+1.513
(optimum)		318	14.21	2.45	−2.37		
		338	16.02	4.00	−3.90		
	100	298	55.01	1.22	−0.50	+3.32	+0.935
		318	57.98	1.38	−0.85		
		338	64.04	1.78	−1.62		
	500	298	152.11	0.44	2.05	+1.68	+0.746
		318	170.05	0.52	1.75		
		338	184.97	0.59	1.50		
	1000	298	185.02	0.23	3.67	+0.61	+0.622
		318	209.98	0.27	3.50		
		338	225.02	0.29	3.48		
pH ~ 10	20	298	13.00	1.86	−1.53	+2.53	+0.873
(natural/non-adjusted)		318	13.41	2.03	−1.87		
		338	14.04	2.33	−2.38		
	100	298	51.01	1.04	−0.10	+1.72	+0.603
		318	52.95	1.13	−0.32		
		338	56.04	1.27	−0.68		
	500	298	135.01	0.37	2.46	+1.19	+0.571
		318	145.07	0.41	2.37		
		338	160.02	0.47	2.12		
	1000	298	149.96	0.18	4.30	+1.02	+0.503
		318	170.07	0.20	4.19		
		338	200.02	0.25	3.90		

Another interesting finding was that ΔH^0^ and ΔS^0^ showed a decrease with increasing initial concentration and amount adsorbed. This effect could be attributed to: (1) The energetic heterogeneity of the surface; and (2) possible dye-dye interactions. The dependence of heat of adsorption with surface coverage is usually observed to display the adsorbent-adsorbate interactions followed by adsorbate-adsorbate interactions [42]. At low concentrations, hence at low Q_e_, the adsorption sites having the highest affinity for dyes are occupied first, while then, with increasing Q_e_, the remaining sites with lower affinity are progressively occupied. The variation of heat of adsorption with surface loading can be also attributed to the possibility of lateral interactions between dye molecules.

### 3.6. Effect of Dosage of Adsorbent (Real Samples)

**Figure 7:**
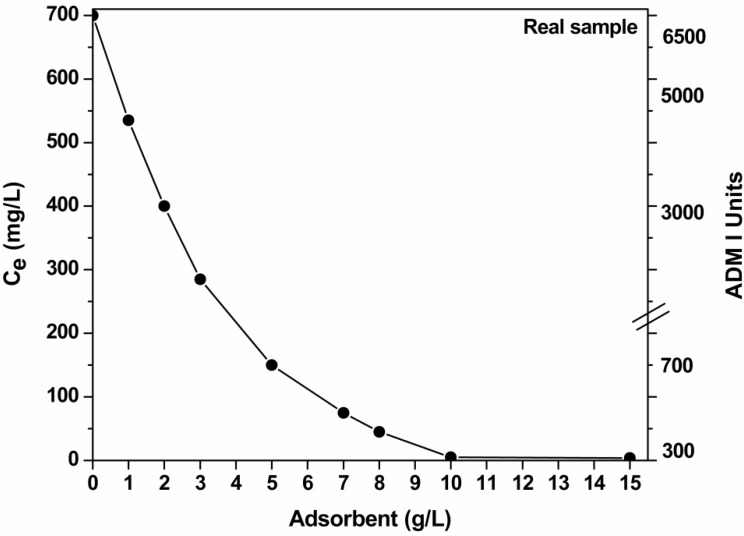
Effect of mass of adsorbent (COF) on decolorization of real textile effluents (pH = free, 700 mg/L total dye concentration; 1 g/L adsorbent; T = 25 °C; 60–140 rpm; 24 h contact).

Figure 7 illustrates data from real textile effluents by varying the mass of adsorbent. The pH of the textile effluent was measured (pH ~ 10) and left without adjustment. The total residual dye content was expressed both in concentration (mg/L) and ADMI units [28]. Given the high dye content of real sample and the ADMI technology, the reduction in ~300 ADMI units is a very sufficient level [1,6,7,28]. It is obvious that increasing the adsorbent’s dosage, the decolorization is improved. For 10 g/L as dosage, a full decolorization is observed.

### 3.7. Desorption–Reuse

Before the investigation of reuse, desorption experiments were carried out to find the optimum pH-desorption conditions. The contrast phenomenon from the adsorption is observed (data not shown), presenting the highest desorption percentage in alkaline conditions (92%). It is generally surprising to get a good adsorption and desorption at the same pH, given the adsorption experiments for real effluents were performed at natural pH (~10). However, there was a significant difference between the two processes with respect to the ionic strength: adsorption was realized in the presence of ~1 mol/L NaCl, while desorption in its absence. The high electrolyte content generates high osmotic pressure in the bulk and causes more sodium ion penetration in the inner of adsorbent, which eventually helps/favors dye molecule uptake. In the contrary, the absence of electrolyte reverses the mass transfer from the solid phase to the bulk [4].

In dyeing technology many additives are used, such as salts and surfactants, which can either accelerate or retard dye adsorption processes. Sodium chloride which is often used as a stimulator in dyeing processes can act in dual mode: (1) It may screen the electrostatic interaction of opposite charges in adsorbents and the dye molecules, and an increase in salt concentration could decrease the amount of dye adsorbed; (2) it may enhance the degree of dissociation of the dye molecules and facilitate the amount of pollutant adsorbed. The ionic strength is thus another important factor in the dye adsorption process. In the case of real samples tested, the adsorption mechanism is possibly influenced by the presence of high ionic strength (~1 M NaCl). The latter cannot be avoided. The effect of ionic strength can be seen from the dye content removal, which is higher in synthetic sample than in real (where the salinity is the real used). The ionic strength of dye solution has to be inevitably accepted either positively or negatively. It is not a factor that can be changed or if changed, the cost is high. It is thus ignored in the current study, giving a more realistic and practical impression of the work.

**Figure 8:**
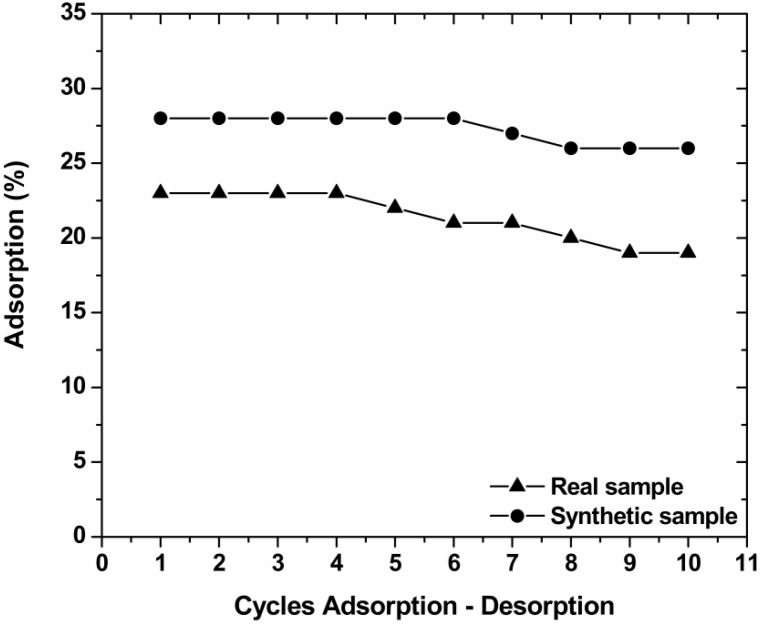
Cycles of adsorption-desorption for the reuse of COF for decolorization of real and synthetic textile effluent (pH_ads_ = free; pH_des_ = 10; 1 g/L adsorbent; T = 25 °C; 140 rpm; 24 h contact).

To investigate the reuse of the materials used, sequential adsorption-desorption experiments in batch mode were conducted for 10 cycles. Figure 8 shows that the reduction in adsorption percentages from the 1st to 10th cycle was 4% for COF. This decrease occurred can be attributed to several reasons as: (1) A progressive saturation of the active adsorption sites/groups by dye molecules; (2) a degradation of material due to extreme pH conditions; (3) a progressive blocking of the active sites of the adsorbent by possible impurities caused a slight decrease in the adsorption potential.

## 4. Conclusions

The decolorization of real textile wastewaters was studied with coffee wastes (COF) as low-cost adsorbents. The optimum pH found after adsorption experiments was pH = 2 for synthetic dyeing effluents, while experiments in free pH (non-adjusted) were carried out for real effluents. Equilibrium data were fitted to the Langmuir, Freundlich and Langmuir-Freundlich (L-F) models. The best correlation was for the L-F model (R^2^ ~ 0.999). The calculated maximum adsorption capacities (Q_max_) for total dye removal at 25 °C was 241 mg/g (pH = 2) and 179 mg/g (pH = 10). Thermodynamic analysis was realized, wherein the positive values of ΔH^0^ suggest the endothermic nature of the process, the negative values of ΔG^0^ suggest that the process is spontaneous with high preference for dye molecules, and the positive values of ΔS^0^ show the increased randomness at the solid/liquid interface. Kinetic data were fitted to the pseudo-first, -second and -third order model. The best correlation was for pseudo-first order equation. The optimum desorption pH found was pH = 10, and after 10 cycles of reuse, the reduction in adsorption percentages from the 1st to 10th cycle was approximately 4% for COF adsorbents. In increasing the mass of COF adsorbents, a strong increase in total dye removal occurred.
